# In vitro thyroperoxidase inhibition assessment by LC-ICP-MS-based L-tyrosine iodination assay: comparison with Amplex Ultrared assay and its modifications

**DOI:** 10.1007/s00204-025-04258-y

**Published:** 2026-01-14

**Authors:** Runze Liu, Jiří Novák, Jan Kuta, Marie Smutná, Klára Hilscherová

**Affiliations:** Faculty of Science, RECETOX, Masaryk University, Kamenice 753/5, pavilion A29, 625 00 Brno, Czech Republic

**Keywords:** Thyroid, Endocrine disruption, Peroxidation, Cross-species, Tyrosine iodination

## Abstract

Disruption of the thyroid hormone (TH) system by environmental chemicals poses significant risks to human and wildlife health. Reliable in vitro assays are essential for assessing thyroid peroxidase (TPO) inhibition, which disrupts a key step in thyroid hormone (TH) synthesis, while adhering to 3R principles. This study aimed to compare the sensitivity and specificity of two types of in vitro assays, tyrosine iodination (Tyr-I) detected by HPLC-ICP-MS and Amplex UltraRed (AUR) and its modifications, using human HEK-TPOA7 cells and rat thyroid microsomes. It involved the evaluation of TPO inhibition by 21 chemicals (concentration range 0.002–200 µM) from diverse use categories, including industrial pollutants, pesticides, and pharmaceuticals. TPO-inhibition potential was indicated for 14 compounds. The Tyr-I assay, which measures the conversion of L-tyrosine to monoiodotyrosine, demonstrated superior sensitivity by capturing both the peroxidation and iodination steps of TPO activity, with effective concentrations for some human exposure-relevant chemicals (benzophenone 2, resorcinol) in the 15–74 nM range confirmed with both human and rat TPO sources. In contrast, the AUR assay detects only the peroxidation step, limiting its ability to fully assess TPO inhibition. The inclusion of sodium iodide (NaI) in the AUR assay significantly enhanced its sensitivity (though it was still lower than in Tyr-I assay), while adding L-tyrosine together with NaI did not. The study documents the advantages and limitations, as well as the application and interpretation potential of the different assays´ variants. It provides valuable information and scientifically sound methodology to support the development of efficient testing strategies for the assessment of thyroid hormone system-disrupting chemicals.

**Supplementary Information:**

The online version contains supplementary material available at 10.1007/s00204-025-04258-y.

## Introduction

Humans and animals are continuously exposed to a wide array of chemicals from both natural and synthetic sources. These substances often coexist in complex mixtures, comprising both parent compounds and their degradation products, which can interfere with biological processes and lead to adverse health effects, even at low doses. Among these, endocrine-disrupting chemicals (EDCs) are particularly concerning because they can interfere with hormonal regulation at low concentrations, potentially disrupting homeostasis, growth, metabolism, and other critical physiological functions (Keane et al. 2015). One of the less-explored but highly significant targets of EDCs is the thyroid hormone system (THS), which plays a fundamental role in early brain development, energy metabolism, and growth (Street et al. 2018).

Disruptions in THS have been linked to a range of adverse outcomes in both wildlife and humans. In children, exposure to thyroid hormone system-disrupting compounds (THSDCs) has been associated with neurodevelopmental impairments, including cognitive deficits, behavioral disorders, and reduced IQ (Murthy and Murthy 2012). Additionally, studies in both rats and humans have suggested a connection between thyroid dysfunction and neurological disorders (Kakked et al. 2013; Stoica et al. 2007), as well as metabolic and cardiovascular disorders (Murthy and Murthy, 2012). Human exposure to these chemicals occurs through various sources, including personal care products, consumer goods, plastics, and children’s toys (Giulivo et al. 2016). In aquatic ecosystems, THS disruption in vertebrates has been linked to multiple adverse effects, such as increased larval mortality, impaired gonadal development, reduced reproductive success, disrupted metamorphosis in amphibians, and abnormal swimming behaviors in fish (Noyes et al. 2019; Nugegoda & Kibria 2017). Given the widespread exposure and severe implications of THSDCs, it is critical to improve our understanding of their effects and develop strategies to mitigate their impact on both human health and the environment.

Despite the urgent need to assess whether specific chemicals interfere with THS function, current testing methods remain limited. Existing guidelines, such as those established by the Organisation for Economic Co-operation and Development (OECD), primarily rely on animal testing to evaluate the effects of THSDCs (Bernasconi et al. 2023; Holbech et al. 2020; Knapen et al. 2020; Vergauwen et al. 2024). However, these approaches are not only time-consuming and ethically contentious but also insufficient for rapidly assessing large numbers of chemicals. Consequently, there is a pressing need to develop and validate in vitro bioassays that focus on key mechanisms of THSDC action while reducing reliance on animal models. The Adverse Outcome Pathway (AOP) network for thyroid hormone system disruption, as proposed by Noyes et al. (2019) and Haigis et al. (2023), offers a promising framework for bridging this gap by linking molecular targets to broader adverse outcomes, thereby improving regulatory decision-making. In recent year, there have been major international efforts on OECD level as well by the European Union Reference Laboratory for Alternatives to Animal Testing (EURL ECVAM) and several EU projects to establish and (pre)validate THSD-related assays for potential inclusion in OECD testing guidelines, following the OECD’s 2014 framework for assessing thyroid hormone signaling disruption (Bernasconi et al. 2023; OECD 2014).

A key molecular initiating event (MIE) within the AOP network is the inhibition of thyroperoxidase (TPO), an essential enzyme in thyroid hormone synthesis. TPO catalyzes three critical reactions: (1) the oxidation of iodide to iodine using hydrogen peroxide, (2) the iodination of tyrosine residues within thyroglobulin to form monoiodotyrosine (MIT) and diiodotyrosine (DIT), and (3) the coupling of iodinated residues to generate the thyroid hormones thyroxine (T4) and triiodothyronine (T3) (Ruf & Carayon 2006). Given the central role of TPO in TH biosynthesis, its inhibition serves as one of the key indicators of TH synthesis disruption across diverse vertebrate groups (Haigis et al. 2023).

Several in vitro assays have been developed to assess TPO inhibition. The U.S. Environmental Protection Agency’s (EPA) ToxCast program employed the fluorescence-based Amplex UltraRed (AUR) TPO assay to screen over 1000 chemicals for TPO inhibition using ex vivo rat thyroids as the enzyme source (Friedman et al. 2016). More recently, human TPO-overexpressing cell lines have been used in similar AUR assays (Dong et al. 2020, 2024; Liu et al. 2024). Additionally, alternative fluorescence-based assays using Guaiacol (GUA) as a substrate have been employed to measure TPO inhibition (Jomaa et al. 2015; Schmutzler et al. 2007). Some studies have utilized luminescence-based assays involving luminol and hydrogen peroxide to assess TPO activity (Dong et al. 2020; Godlewska et al. 2018; Jomaa et al. 2015), though recent findings show that these methods may lack specificity for TPO activity (Liu et al. 2024). Notably, most existing assays focus solely on the peroxidation step without accounting for iodination reactions, which are equally crucial in TH synthesis.

Two recent studies have attempted to address this gap by developing in vitro assays that quantify both peroxidation and iodination steps using liquid chromatography-tandem mass spectrometry (LC–MS/MS) and ultra-performance liquid chromatography-mass spectrometry (UPLC-MS/MS), respectively (Tater et al. 2021; Reinen et al. 2024). These assays measure TPO activity by detecting MIT formation from L-tyrosine in the presence of potassium iodide (KI) and hydrogen peroxide (H₂O₂). However, Tater et al. (2021) relied on ex vivo rat thyroid microsomes, which required the use of animals as the source of TPO enzyme. To date, only a single study by Reinen et al. (2024) has successfully developed a complex human TPO activity assay fully compliant with 3R principles (Replacement, Reduction, and Refinement in animal research) using an in vitro source of TPO. This highlights the need for further development and characterization of non-animal in vitro methods that comprehensively assess multiple human TPO-catalyzed reactions.

Our study is part of the recently completed ERGO project, funded under the EU’s Horizon 2020 program, which aimed to improve the testing of THSDCs. ERGO focused on developing an AOP-based network for thyroid hormone disruption, integrating data from multiple vertebrate species to improve biomarkers and screening methods for assessing both human and environmental health risks (Holbech et al. 2020). These efforts aligned with the EURL ECVAM validation study, which sought to accelerate the approval of a suite of THSD-relevant mechanistic in vitro assays, including those targeting TPO inhibition (Bernasconi et al. 2023). An overview of available methods for THSDCs identification and their validation status has been published recently (Vergauwen et al. 2024).

Building on our prior work (Liu et al. 2024), this study critically evaluates the performance of the tyrosine iodination (Tyr-I) assay, which quantifies the conversion of L-tyrosine to MIT catalyzed by TPO using high-performance liquid chromatography coupled with inductively coupled plasma mass spectrometry (HPLC-ICP-MS). Our results, based on TPO lysates derived from an in vitro human cell model overexpressing TPO (HEK-TPOA7), are compared directly with those obtained from the AUR assay, which only assesses the peroxidation step. This study represents the first direct, side-by-side comparison of TPO inhibition using peroxidation and iodination-based assays. By incorporating key methodological refinements, such as the addition of sodium iodide and L-tyrosine into the AUR assay, we aim to improve its physiological relevance. Ultimately, this comparative analysis provides critical insights into the strengths and limitations of current TPO inhibition assessment methods and brings crucial information for future advancements in thyroid hormone disruption research and assessment.

## Material and methods

### Chemicals

In this study, a set of 21 chemicals was tested (Tables 1, S1), which were chosen within the ERGO H2020 project (Holbech et al. 2020) to address diverse THSD modes of action and they represent a wide range of chemical categories, such as drugs, pesticides, industrial chemicals, environmental pollutants, personal care and consumer products, and cleaning agents like disinfectants and antiseptics (Table S1). We obtained these compounds from Sigma-Aldrich in high purity (≥ 98%). More information about how these compounds were selected can be found in our previous paper (Liu et al. 2024).

**Table 1:** Characterization of inhibition effects expressed as IC_50_ (µM; ±  standard deviation) of tested chemicals detected by AUR or tyrosine iodination assay in the current study in comparison with available previously reported data

		AUR assay			Tyrosine iodination assay				AUR assay				
		Current study					Reinen et al. (2024)	Tater et al. (2021)	Dong et al. (2020)^a^	Dong et al. (2020)^a^	Dong et al. (2024)^a^	Friedman et al. (2016)^a^	ToxCast (US EPA 2024)^e^
Detection		fluorescence	fluorescence	fluorescence	LC-ICP/MS	LC-ICP/MS	LC-MS/MS	LC-MS/MS	fluorescence	fluorescence	fluorescence	fluorescence	fluorescence
Modification			NaI	NaI + Tyr									
Abbreviations	TPO source	HEK-TPOA7	HEK-TPOA7	HEK-TPOA7	HEK-TPOA7	Rat microsomes	FTC-238-hrTPO	Rat microsomes	CHO-TPO	LentiX-TPO	LentiX-TPO	Rat microsomes	Rat microsomes/(hTPO)^f^
	Chemicals												
AMP	Ampicillin	NA	≤ 40%	NA	12.9 (± 4.1)	-	173/258^b^						
BP2	Benzophenone-2	0.538 (± 0.060)	0.586 (± 0.14)	0.478 (± 0.015)	0.0221 (± 0.0046)	0.0235 (± 0.0064)	0.0256/0.0326^b^				2.53 (± 5.5)	0.17	0.272
BPA	Bisphenol A	7.41 (± 1.7)	9.73 (± 4.9)	15.4 (± 1.5)	0.278 (± 0.093)	1.24 (± 0.40)					0.315/0.595^c^		6.70
CBZ	Carbamazepine	NA	NA	NA	NA	-							
DBP	Dibutylphthalate	NA	NA	NA	NA	-					99.0 (± 3.1)		
DON	Deoxynivalenol	NA	NA	NA	NA	-							
ETU	Ethylene thiourea	NA	≤ 23%	NA	1.42 (± 0.50)	-	1.55/1.21^b^				34.8^c^	7.8	7.30
IOP	Iopanoic acid	NA	≤ 41%	≤ 27%	≤ 39%	-							
MMI	Methimazole	1.49 (± 0.32)	1.03 (± 0.21)	0.825 (± 0.19)	0.484 (± 0.10)	1.01 (± 0.35)	0.384 [0.365 − 0.393]^b^		0.49	0.23	0.08 (± 0.04)	0.06	0.102 /(0.13)^f^
PCL	Perchlorate	NA	NA	NA	NA	-	NA/NA^b^						
PFOA	Perfluorooctanoic acid	NA	≤ 21%	NA	NA	-							NA
PFOS	Perfluorooctane sulfonate	NA	≤ 39%	NA	≤ 25%	-	56.5/52.4^b^						
PTU	6-propylthiouracil	6.21 (± 1.3)	5.56 (± 2.3)	7.03 (± 1.8)	1.47 (± 0.35)	1.61 (± 0.25)	1.61 [1.21 − 1.94]^b^	3.37 (± 0.58)	4.9	10.4	2.56^c^	0.23	0.295
RSC	Resorcinol	0.361 (± 0.13)	0.394 (± 0.13)	0.319 (± 0.017)	0.0149 (± 0.0017)	0.0739 (± 0.024)	0.0130/0.0167^b^		0.05	0.06	0.52 (± 5.2)	0.025	0.0475
SA	Salicylic acid	NA	NA	NA	NA	-							
SMX	Sulfamethoxazole	≤ 23%	115 (± 17)	≤ 20.3%	16.9 (± 6.6)	2.91 (± 0.90)							
T3	3,3′,5-Triiodo-L-thyronine	NA^h^	3.32 (± 0.71)	5.79 (± 0.14)	1.72 (± 0.69)	5.34 (± 3.4) ^g^							
T4	3,3′,5,5″-Tetraiodo-L-thyronine	NA	NA	NA	≤ 27%	-							
TBBPA	Tetrabromobisphenol A	9.16 (± 1.4)	5.28 (± 2.5)	7.37 (± 1.6)	1.95 (± 0.60)	7.31 (± 4.6)	0.559/0.900^b^						21.8
TCS	Triclosan	199 (± 22)	27.2 (± 6.8)	183 (± 14)	5.92 (± 2.0)	17.0 (± 3.9)	3.58/3.60^b^		> 253	> 253	88.2 (± 6.29)	48	52.16^d^
TPP	Triphenyl phosphate	NA	NA	NA	NA	-							

Regarding our results, “NA”—inactive (< 20% inhibition) up to the highest tested concentration specified in the methodology and Table S1. “-” not tested. If there was a statistically significant inhibition between 20 and 50%, the average of the maximum inhibition values across all independent experiments is included (e.g. ≤  40%). Number of independent replicates in the current study, for active chemicals (inhibition  ≥  20%) n ≥  3 (n ≥  2 for rat microsomes), and inactive chemicals (NA), n  ≥  2

^a^AC_50_ represents the concentration of the chemical at which 50% of the maximum detected inhibition is observed, where the maximum inhibition is determined relative to the highest tested concentration even if it does not reach 100%. Thus, the AC_50_ value is often lower than the corresponding IC_50_ value. In contrast, IC_50_ corresponds to the concentration at which 50% inhibition is observed on the Y-axis, where 100% represents no inhibition (full activity)

^b^From two independent experiments. 30 blinded chemicals were tested in at least two valid and independent experiments. Some chemicals were tested more than twice; the range provides data from five independent experiments

^c^Data from an independent assay run using anonymized test samples analysis

^d^IC_20_ value

^e^Data from the ToxCast recalculated to IC_50_ values from the provided dose–response data

^f^human TPO transfected into the HEK293T cell line

^g^the IC_50_ value extrapolated; highest tested concentration 5 µM

^h^T3 marked NA as the inhibition did not reach 20% (caused a mean 18% inhibition at the maximal tested concentration of 10 µM)

### Cell culture

The HEK-TPOA7 cell line was derived from the HEK293T cell line, a human SV40-transformed embryonal kidney immortalized cell line obtained from ATCC, by transfection with hTPO as described previously (Liu et al. 2024). The cells were cultured in DMEM High Glucose media supplemented with 10% FBS. The cells were exposed to hematin (1 µg/mL) together with HEPES solution (Sigma-Aldrich; pH to 7.3; final concentration in medium 10 mM) for at least 2 days before the cell harvesting for lysate preparation (Schmutzler et al. 2007).

### TPO enzyme preparation

Human cell lysates were prepared from the cultured cell lines as the whole cell extract as described previously (Jomaa et al. 2015). Briefly, the cells were washed with phosphate-buffered saline (PBS), scraped, resuspended in PBS, and centrifuged for 5 min at 150 rcf. The supernatant was discarded, and the pellet was lysed with 0.1% m/v sodium deoxycholate in PBS and incubated on ice for 20 min. Lysed cells were centrifuged for 5 min at 12,000 rcf to extract the soluble protein fraction. The cell lysates were kept at − 80 °C for longer-term storage.

Rat thyroid microsomes were prepared following the procedure described previously (Abas & Luschnig 2010), and the use of rats was approved by the Institutional Animal Ethics Committee (MSMT 2634/2019–5). Briefly, thyroid glands were collected from twelve-week-old female Wistar rats. The collected thyroids were stored at − 80 °C. Thyroids from 20 rats were pooled together for microsome isolation. Pooled thyroid glands were thawed on ice and cut into fine pieces using a sterile surgical blade in PBS containing a protease inhibitor cocktail (Roche, Switzerland), followed by homogenization using Potter–Elvehjem PTFE assembly. Thyroids from 20 animals were homogenized in approximately 3 mL of PBS. The homogenized tissue suspension was passed through a 23G hypodermic needle 15–20 times. An equal volume of 50% sucrose was added to the homogenized tissue suspension, and the tubes were centrifuged at 600 rcf for 5 min at 4 °C. The pellet was discarded, the supernatant was collected in fresh tubes, and an equal volume of sterile water was added. Homogenized tissue suspension was distributed equally (in 250 µL aliquots) in multiple tubes and centrifuged at 20,000 rcf for 2 h at 4 °C. The supernatant was discarded. The microsome pellet was pooled and resuspended in 1 mL 5% glycerol in PBS, and aliquots of microsomes were stored at − 80 °C until further use.

The Bio-Rad DC-protein kit (Bio-Rad, Czech Republic) was used to measure the protein content in the cell lysates and microsomes following the manufacturer’s protocol.

### TPO activity assessment by tyrosine iodination assay

The Tyr-I assay has been based on work by Tater et al. (2021) with several modifications. The reaction was conducted in amber glass vials with a reaction mixture final volume of 100 μL per vial containing 20 μL cell lysate (protein concentration 0.6 mg/mL, final concentration 0.12 mg/mL) or of rat microsomes (final concentration 0.04 mg/mL), 15 μL 3300 μM L-tyrosine (L-Tyr; final 500 μM), 20 μL 2500 μM NaI (final 500 μM), and 25 µL of PBS with the test chemical added in 1 µL of a 100-times concentrated stock solution in MeOH (1% MeOH final concentration). After 5 min incubation, the reaction was initiated by adding 20 μL of 200 μM H_2_O_2_ (final 40 μM) and incubated at 37 °C. The reaction was stopped after 60 min by adding 900 µL of 50% MeOH. The samples were immediately loaded into an auto-sampler tray, and 10 µL was injected into HPLC-ICP-MS. A set of control assays with no active TPO (denatured HEK-TPOA7 cell lysate and non-transfected HEK293T cell lysate), with PBS instead of cell lysate or rat microsomes (a protein-free control), without H_2_O_2_, NaI, or L-Tyr, were done to verify the assay performance.

The concentrations of iodine-containing compounds (iodide and MIT, DIT, and T4) were quantified by high-performance liquid chromatography (Agilent 1260 Infinity II HPLC in bio-inert design or Agilent 1100 HPLC) in combination with inductively coupled plasma mass spectrometry (Agilent 8900 ICP-MS/MS or Agilent 7700 × ICP-MS). Analytes were separated on an Agilent Eclipse XDB-C18 reversed-phase column (4.6 × 150 mm, 5 µm). The mobile phase flow rate was set to 1 mL/min, the oven temperature was maintained at 25 °C, and 10 µL of the sample was injected into the column. The mobile phase for the gradient separation contained 0.1% solution of trifluoroacetic acid in deionized water (channel A) and 0.1% solution of trifluoroacetic acid in methanol (channel B). The gradient program in terms of mobile phase B was as follows: 0 – 2 min linear ramp from 35 to 95%, 2–3 min at 95%, and 3–5 min at 35%. The ICP-MS/MS system was equipped with a quartz MicroMist nebulizer, Scott-type double-pass quartz spray chamber, 1.5 mm ID quartz plasma torch, platinum interface cones, and brass skimmer base. ICP was operated at 1600 W, the spray chamber temperature was adjusted to − 5 °C, and the nebulizer gas flow rate was set to 0.63 mL/min. Option gas (20% O_2 _in Ar) was used for the oxidation of organic matter in aerosol with a flow rate of 20% of the carrier gas flow rate. Analyte detection was performed on ^127^I^+^ isotope with a dwell time of 1.5 s.

### TPO activity assessment by AUR assay with modifications

The standard AUR assay has been conducted as described previously using a cell lysate (Liu et al. 2024; Paul et al. 2014). Briefly, the assay was performed in black 96-well plates (Greiner Bio-One, Austria) with a final volume of 100 μL per well containing 50 μL cell lysate with protein content of 0.12 mg/mL (final 0.06 mg/mL), 20 µl PBS with 1 µL of the test chemical in MeOH (1% MeOH final concentration). The reaction was initiated by adding 25 μL of 100 μM (final 25 μM) Amplex UltraRed solution in PBS and 5 µL of 800 μM H_2_O_2_ (final 40 µM). After 30 min incubation at 37 °C in the dark, the fluorescence endpoint was measured on the plate reader Synergy MX (Biotek Agilent, Stevens Creek, USA) at 544 nm excitation/590 nm emission. For the blank in the standard AUR assay, 50 μL of cell lysate was replaced with 50 μL of PBS.

### Modification of the AUR assay by adding L-tyrosine and/or iodide

We tested modifications to the AUR assay, incorporating sodium iodide and L-tyrosine, which are components of the Tyr-I assay with better sensitivity, and are also involved in the in vivo conditions.

First, several iodide concentrations were tested to study the impact of its addition on AUR assay performance, and 20 µL PBS with 1 µL of the test chemical in MeOH (1% MeOH final concentration) was replaced by 20 µL of NaI in PBS at different concentrations (final 37.5, 75, 150, 300, 500 µM). In parallel, a corresponding control series was performed where the cell lysate was replaced with the same volume of PBS or TPO-free cell lysate (heat-inactivated HEK-TPOA7 cell lysate, boiled at 95 ⁰C for 10 min or lysate from non-transfected HEK293T cells; final protein content 0.06 mg/mL).

The final modified setup was based on 500 µM iodide, corresponding with the Tyr-I assay. Thus, the 20 µL PBS with 1 µL of the test chemical in MeOH (1% MeOH final concentration) used in the unmodified assay was enriched with 2500 µM NaI (final 500 µM) or 2500 µM NaI (final 500 µM) and 2500 µM L-tyrosine (final 500 µM) in the NaI and NaI + L-tyrosine-enriched setups, respectively. For the control, 50 μL TPO-free cell lysate was used as described above. In the experiments assessing TPO inhibition of the model chemicals, heat-inactivated HEK-TPOA7 cell lysate was used as the TPO-free cell lysate background control (see Fig. S4).

### Data evaluation

For the data analysis, GraphPad Prism™ (GraphPad Software, San Diego, USA) was used for curve-fitting and IC_50_ calculation. The IC_50_ levels were calculated only in cases where the inhibition was statistically significant compared to adequate solvent control. The data were blank corrected i.e. the signal of the background control (average fluorescence intensity or MIT level, in case it exceeded LOQ level in TPO-free protein control exposure variants for AUR or Tyr-I assay, respectively) was subtracted from the TPO-containing exposure variants (fluorescence intensity or MIT level) and then normalized to the signal in the solvent control exposure variant (corresponding to uninhibited 100% TPO activity). Fold change of signal intensity in the AUR assay and its modifications was calculated by dividing the signal from cell lysate-containing wells by the signal from the background (average of wells with TPO-free protein control). Tested chemicals were considered active if they caused at least 20% inhibition compared to the control.

## Results and discussion

Our study contributes to international initiatives aimed at establishing standardized methodologies for evaluating thyroid hormone system disruption, with a particular focus on thyroid peroxidase (TPO), a key enzyme in TH biosynthesis. TPO inhibition represents a critical mechanism of action for chemicals associated with adverse effects across many vertebrate species, and its importance is well-documented by the Adverse Outcome Pathway (AOP) framework (Haigis et al. 2023; Hornung et al. 2015; Noyes et al. 2019). Well-characterized and validated methods that are in vitro-based and do not require any ex vivo material are needed to enable high-throughput assessment of this endpoint across a wide range of chemicals and environmental exposure mixtures (Vergauwen et al. 2024). Therefore, advancing the development, characterization, and validation of non-animal in vitro methods is essential to enhance the predictive accuracy of THSDCs assessments and improve regulatory decision-making.

First, the conversion of L-tyrosine (L-Tyr) to monoiodotyrosine (MIT) via tyrosine iodination, catalyzed by TPO, was assessed using a tyrosine iodination (Tyr-I) assay. This assay employed high-performance liquid chromatography coupled with inductively coupled plasma mass spectrometry (HPLC-ICP-MS) for the specific detection of MIT, utilizing human TPO derived from a transfected HEK-TPOA7 cell line (for assay optimization data, see SI Chapter 1 and Figs. S1–S4). The results were then compared to those obtained using the standard Amplex UltraRed (AUR) assay. Next, the Tyr-I assay was also performed using TPO-containing rat thyroid microsomes to investigate the comparability of the results obtained with human and rat TPO. Additionally, the AUR assay was modified by incorporating sodium iodide and L-tyrosine to better align with the reaction conditions of the Tyr-I assay and physiological conditions in vivo. Following these optimizations, we systematically compared the performance of the AUR assay, which assesses only the peroxidation step, with the Tyr-I assay that assesses both the peroxidation and iodination steps catalyzed by TPO.

**Fig. 1:**
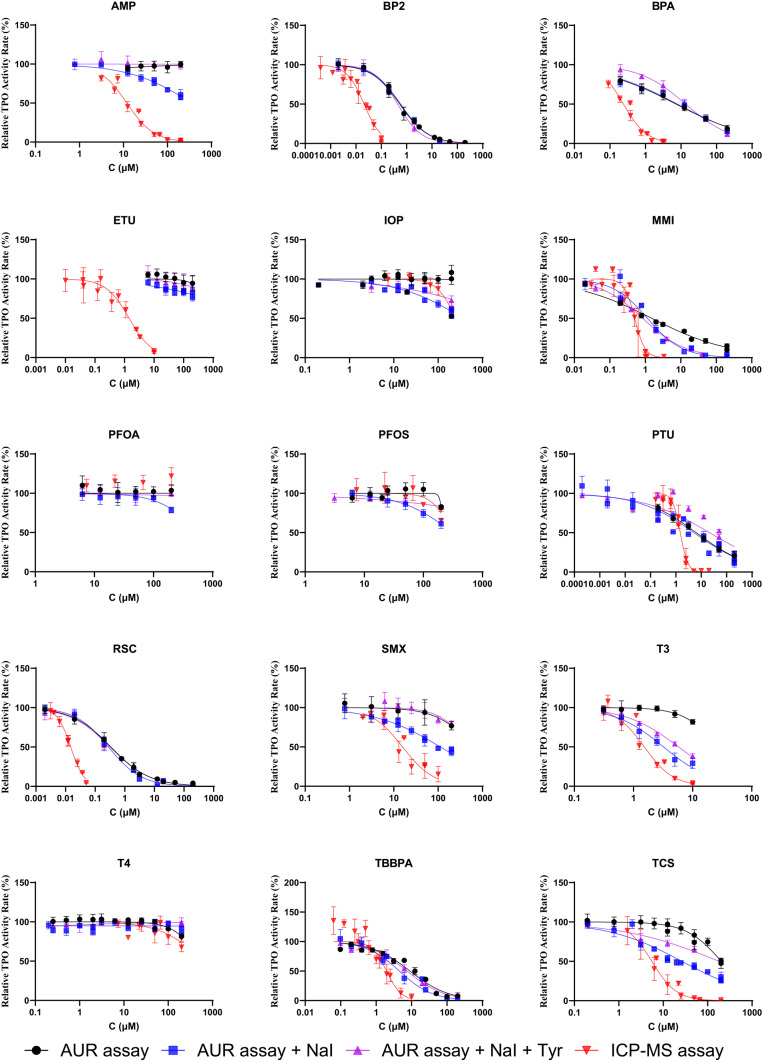
Comparison of dose–response curves for positive chemicals among different assays in human transfected cell line HEK-TPOA7. Relative Effect Rate (%): The signal intensity from the well-containing cell lysate and chemical was background-corrected (average signal intensity of lysate-free wells or boiled lysate wells), then normalized to the solvent control (100% activity). Data from at least three independent experiments for each exposure variant

The data are summarized in Figs. 1, S5, and S6, which show the dose–response curves of chemicals with and without detectable TPO inhibition effects from all assay variants. Table 1 presents the IC_50_ values and mean inhibition rates for all tested chemicals obtained from the assays. “NA” indicates non-active chemicals, defined as those causing less than 20% inhibition up to the greatest tested concentration listed in Table S1.

### Tyrosine iodination assay

In the current study, we focused on the conversion of L-Tyr to MIT in the Tyr-I assay, consistent with previous work to ensure comparability (Reinen et al. 2024). This reaction yielded significantly more product than the conversion of L-Tyr to DIT, which was often near or below the limit of quantification. The detection of produced MIT enabled sensitive assessment of L-Tyr iodination inhibition (Fig. S1). The other steps in thyroid hormone synthesis, such as MIT to DIT and DIT to T4, were not the focus of this paper. Although their products were detectable, they were mostly close to the limit of quantification (Fig. S3). A set of control experiments has confirmed that MIT synthesis requires all essential components: L-tyrosine, iodide, and hydrogen peroxide as the TPO substrates (data not shown). The controls with denatured TPO-containing homogenate and/or homogenate from non-transfected HEK293T cells need to be always included in the assay to ensure the spontaneous non-enzymatic MIT production does not affect the assay performance and provide a background TPO activity control (see results and more discussion on this topic in Supplementary Materials (SM) Chapter 1). Analytes were quantified using an external calibration method with quantification limits of 16 and 4.6 nM for MIT and DIT in the reaction mixture, respectively. Quantification limits were comparable to or lower than those reported in studies based on similar assays with LC–MS/MS detection by Tater et al. (2021) (0.547 and 0.273 nM after dilution for the analysis, which corresponds to 23.5 and 11.7 nM in the reaction mixture, for MIT and DIT, respectively) and Reinen et al. (2024) with LOQ for MIT at 76.1 nM in the reaction mixture. The determination of MIT and DIT by HPLC-ICP-MS serves as a valuable alternative method to more commonly used LC–MS/MS detection, as has been previously demonstrated by the determination of MIT and DIT, e.g., in seaweed (Romarís-Hortas et al. 2013) or dog food (Wilson et al. 2016). For the information on assay optimization, see SM.

The results from the Tyr-I assay based on the human-transfected HEK-TPOA7 cell line show that AMP, BP2, BPA, ETU, MMI, PTU, RSC, SMX, T3, TBBPA, and TCS inhibited MIT production by more than 50%, which enabled the quantification of IC_50_ values. BP2 and RSC showed the highest inhibition potency (IC_50_ 0.022 and 0.015 µM, respectively). BPA and MMI also elicited effects with IC_50_ values in the sub-micromolar range (0.278 and 0.484 µM, respectively). On the other hand, AMP and SMX were less potent inhibitors with the IC_50_ of 12.9 and 16.9 µM. The inhibition induced by IOP, PFOS, and T4 did not reach levels high enough to allow quantification of the IC_50_, although it was greater than 20% and statistically significant compared to the control (Fig. 1, Table 1).

BP2 and RSC were the most potent TPO inhibitors. BP2 is a UV filter used in personal care and industrial products. Human exposure occurs primarily through dermal absorption from personal care products, ingestion of contaminated food and water, and inhalation of indoor dust (Mao et al. 2022). Though less frequently detected than benzophenone 1 and 3, it has been identified in human biological samples, including urine, blood, breast milk, placenta, and amniotic fluid, raising concerns about exposure and potential health risks. Although detection of BP2 was occasionally detected in urine samples based on the European Human Biomonitoring Dashboard, some studies report a maximum BP2 concentration of 336 μg/L (1.36 µM) in urine, much higher than the Tyr-I IC_50_ value detected in our study, while blood levels reached only a maximum of 0.1 μg/L (0.0004 µM) (Mao et al. 2022). In placental tissue, the highest recorded concentration was 8.9 ng/g, confirming placental transfer (Mao et al. 2022). RSC is a versatile chemical extensively utilized across various industries and personal care formulations. In industrial applications, it contributes to the production of rubber products, wood adhesives, flame retardants, UV stabilizers, and dyes. Meanwhile, in personal care, it is frequently found in hair colorants, anti-acne solutions, and exfoliating peels. A Finnish study found RSC in the urine of nearly all individuals from the general population (median: 42 μg/L (0.38 µM), 95th percentile: 1856 μg/L (16.8 µM), maximum 9996 μg/L (90.8 µM)), with women showing higher levels than men (Porras et al. 2018). Hairdressers had similar exposure (median urine levels 78 μg/L (0.79 µM) after-holiday, 44 μg/L (0.40 µM) post-shift), while tire manufacturing plant workers had morning median levels of 114 μg/L (1.04 µM), and a maximum of 3575 μg/L (32.5 µM) in the evening (Porras et al. 2018). In another study with 17 hairdressers, RSC levels ranged from 2.2 to 1824 μg/L (0.020 to 16.6 µM) (Cambrai-Erb et al. 2025). Although the blood levels were probably significantly lower than the detected urine levels, it is likely that they could reach the Tyr-I inhibitory concentration range in the population.

When comparing the results of the Tyr-I assay with those from the unmodified AUR assay, both using the same in vitro hTPO source as reported in our previous study (Liu et al. 2024) and incorporating additional data from an unmodified AUR assay variant, a clear trend emerged. All effective chemicals exhibited stronger inhibition in the Tyr-I assay, as indicated by lower IC_50_ values (Table 1). Seven of the 21 tested chemicals showed no significant inhibition in either assay up to the highest tested concentration (200 µM; Fig. S5). IC_50_ values could be derived for seven chemicals in both assays. Notably, for BP2, BPA, RSC, and TCS, IC_50_ values in the AUR assay were more than 20 times greater than those observed in the Tyr-I assay. While SMX and T3 caused only 23% and 18% mean maximal inhibition, respectively, in the AUR assay, they reached IC_50_ in the Tyr-I assay. Additionally, AMP and ETU produced measurable IC_50_ values in the Tyr-I assay but showed no inhibition in the AUR assay. Unlike in our previous study, PFOA did not elicit any significant inhibition in the TPO-AUR assay up to 200 µM concentration, which is in line with the Tyr-I assay and data from the literature.

With the ≥  20% threshold as the criterion for TPO inhibitor identification, AMP, ETU, IOP, PFOS, T3, and T4 were identified as inhibitors in the Tyr-I assay but not in the unmodified AUR assay (Table 1). These results highlight the superior sensitivity of the Tyr-I assay for the detection and quantification of TPO inhibition. The higher sensitivity of the Tyr-I assay can be attributed to its ability to cover both key steps in TPO activity—peroxidation and iodination—using physiological substrates, thus more accurately reflecting TPO’s enzymatic function. Thus, the Tyr-I assay’s enhanced sensitivity and realistic substrate use make it more reliable for assessing TPO inhibition.

### Tyrosine iodination (Tyr-I) assay using rat microsomes

The rat thyroid microsomes were utilized as TPO sources in the Tyr-I assay to assess the inhibitory effects of a subset of compounds that were detected as human TPO inhibitors by the Tyr-I assay (Table 1 and Fig. S6). The tested chemicals included BP2, BPA, MMI, PTU, RSC, SMX, T3, TBBPA, and TCS, and they all showed strong inhibition of the rat TPO. BP2 and RSC showed the strongest rat TPO inhibition with IC_50_ values of 0.0235 µM and 0.0739 µM, respectively. TCS showed the highest quantified IC_50_ value (17 µM). Thus, BP2 and RSC were identified as the most potent TPO inhibitors using both human and rat TPO sources.

Compared with human TPO (HEK-TPOA7), BP2, MMI, and PTU showed very similar IC_50_ values in the assay variant with rat microsomes (RM). Also, the IC_50_ of TBBPA and T3 were relatively comparable considering their standard deviation. For the T3, the highest tested concentration (5 µM on RM) caused close to 50% inhibition (mean 38%), so the IC_50_ value was estimated by extrapolation to enable comparison with other assay variants (Fig. S6, Table 1). BPA, RSC, and TCS showed higher IC_50_ values with rat TPO, and SMX showed a lower IC_50_ value with the rat TPO, yet the differences were all less than sixfold (within order of magnitude).

In our previous paper, the in silico SeqAPASS analysis documented 82% similarity of the TPO active domain between human and rat, suggesting that findings from studies on one species might be indicative of the effects on the other (Liu et al. 2024). Although the tested set of chemicals was limited in drawing any strong conclusions, the obtained data corresponded to this prediction, as the Tyr-I assay with both TPO sources identified the same range of potencies across the assessed compounds. There were no strong species-specific differences in the potency of TPO inhibition. The results indicate that data from rat TPO may provide relevant qualitative information for human TPO inhibition and vice versa, while there might be some differences in the exact IC_50_ values. Nevertheless, a more extensive set of chemicals (and various batches from different sources of rat microsomes) would need to be tested, including some negative compounds, for definitive conclusions in this regard. Importantly, the Tyr-I assay with an in vitro hTPO source showed mostly comparable or better sensitivity and did not require any ex vivo enzyme source nor preparation of microsomes, which makes it a very good human-relevant option complying with 3R principles. To improve the accuracy and translational relevance of TPO inhibition studies, potential species-specific differences should be considered carefully when translating data across species.

### Comparison with Tyr-I assays from other studies

Reinen et al. (2024) used human TPO-transfected cell lines with the Tyr-I assay, which produced MIT, analyzed by Ultra- and Performance Liquid Chromatography with Tandem Mass Spectrometry (UPLC-MS/MS), while Tater et al. (2021) employed rat microsomes with the Tyr-I assay coupled with HPLC-MS/MS. There was only PTU tested both in the current study and Tater et al. (2021), and its IC_50_ levels corresponded very well between the studies despite potential interspecies differences (1.61 ± 0.25 µM and 3.37 ± 0.58 µM in the current and Tater et al. (2021) study, respectively; Table 1). In the case of Reinen et al. (2024), there were 10 chemicals overlapping with the current study. Among these, only IC_50_ values of PFOS and AMP differed, while the rest were comparable in both studies. The results document the high reproducibility and robustness of the Tyr-I assay (Table 1) even though the assays´ setup differed in the in vitro hTPO overexpressing models used as the enzyme source, concentration of some reagents, as well as the method employed for MIT detection (ICP-MS in our study and LC–MS/MS in Reinen et al. 2024).

### The AUR assay modified with sodium iodide and L-tyrosine

One of the differences between the AUR and Tyr-I assays is the lack of some of the substrates for TPO in the former. AUR assay, per se, is able to detect only TPO’s peroxidation activity, so the substrates for the other activities (iodination and coupling) are usually not included in this assay. On the other hand, even if not necessary, they could indirectly affect the activity of the TPO enzyme. Thus, we have examined how the addition of iodide or iodide with L-Tyr affects the performance of the AUR assay.

**Fig. 2:**
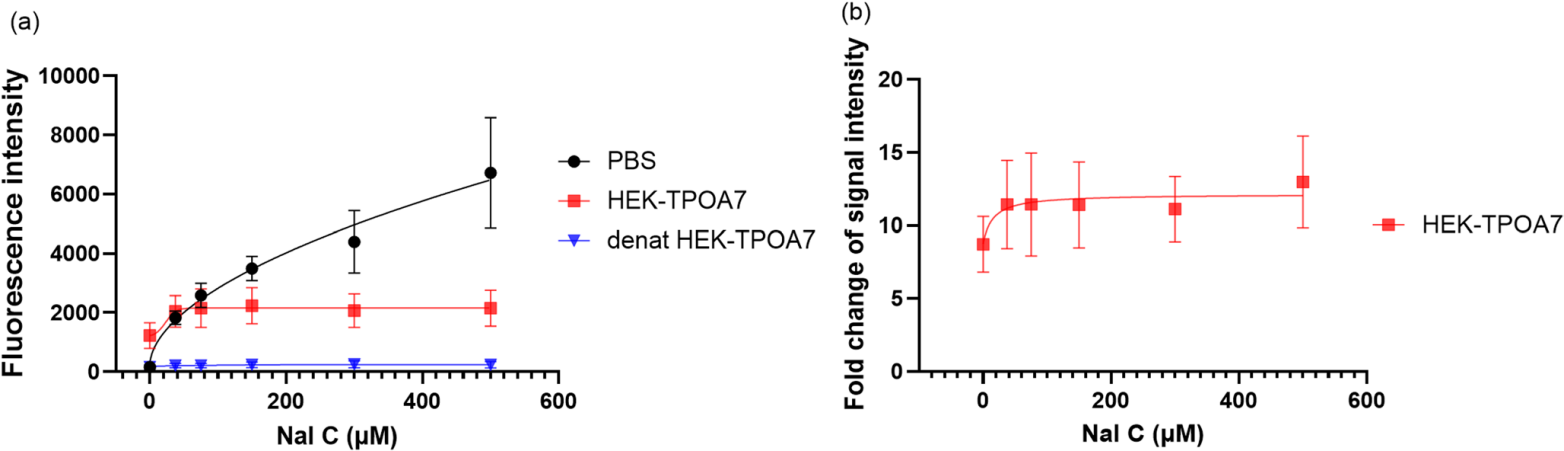
Relative fluorescence intensity (**a**) or fold change in fluorescence intensity for native lysate of HEK-TPOA7 cells to denatured HEK-TPOA7 cell lysate (**b**) of the AUR assay in different NaI concentrations (data from four independent experiments). Denat HEK-TPOA7—heat-inactivated lysate of HEK-TPOA7 cells. After adding NaI, an increase in fluorescence intensity was observed. The PBS group exhibited unexpected fluorescence intensity. In contrast, the denatured lysate group showed fluorescence intensity similar to that of PBS without NaI. **b** Fold change of fluorescence intensity derived by dividing the signal intensity of native HEK-TPOA7 cell lysate by the signal intensity from the control with denatured cell lysate. There was minimal difference in fluorescence intensity across different iodide concentrations

Firstly, the influence of NaI on fluorescence intensity in the AUR assay was studied. Regarding the effect of NaI on the fluorescence signal, Figs. 2a and S4 illustrate that when exposed without TPO (PBS without cell lysate), NaI caused a strong increase in fluorescence intensity, indicating that iodide alone, without the presence of TPO, can catalyze the oxidative conversion of AUR with hydrogen peroxide. In contrast, the variant with NaI and TPO-free protein control, in the form of denatured cell lysate from TPO-expressing cells or native lysate from HEK293T cells without TPO expression, shows a very low fluorescence intensity corresponding to the background signal without NaI, documenting that the presence of TPO-free cell lysate can inhibit this iodide-mediated non-enzymatic AUR oxidation. Both types of TPO-free lysate controls, denatured HEK-TPOA7 and native HEK293T, had the same effect on the fluorescence signal, minimizing the non-enzymatic AUR conversion to the same level as in the blank without NaI (no statistically significant difference, Fig. S4). Both control types showed the same results, so either of the lysates can be used as a background control. Our results, in line with our previous work (Liu et al. 2024), demonstrate that the AUR assay is indeed TPO-specific and is not affected by other peroxidases that are likely present in the HEK293T cell lysate. For practical reasons (a single lysate used across the assay), the denatured HEK-TPOA7 lysate was used as the control across all experiments. Thus, since cell lysate from HEK-TPOA7 was the TPO source in the AUR assay, the fluorescence intensity reflects the TPO-specific peroxide reaction during the test with native cell lysate. Therefore, sodium iodide can be included in the AUR assay, with TPO-free cell lysate used as a blank to minimize and control the non-specific oxidation.

A similar trend was observed for the background non-enzymatic MIT production in protein-free controls in the Tyr-I assay (See SI 1 Tyr-I assay optimization, Fig. S2). Moreover, Reinen et al. (2024) reported that levels of iodide and hydrogen peroxide significantly affect the non-enzymatic MIT formation. In their study, the rate was substantially higher than the enzymatic MIT formation when using high 10 mM iodide in combination with 250 µM hydrogen peroxide. Reducing the potassium iodide concentration to 150 µM mitigated the non-enzymatic MIT formation, while enzymatic MIT formation remained comparable to that observed with 10 mM potassium iodide and was significantly elevated above background levels. The results of the current study document that the presence of cell lysate without TPO activity prevents the non-enzymatic MIT production in the used assay setup (Fig. S2). These findings further underscore the importance of using appropriate controls to account for non-TPO-specific reactions. As has been proposed earlier, the likely explanation of the non-enzymatic production of MIT in the presence of L-tyrosine in the lysate-free variant is the spontaneous formation of iodinating species, such as iodonium ions or hypoiodous acid, in the presence of H₂O₂ (Huwiler et al. 1985).

**Fig. 3:**
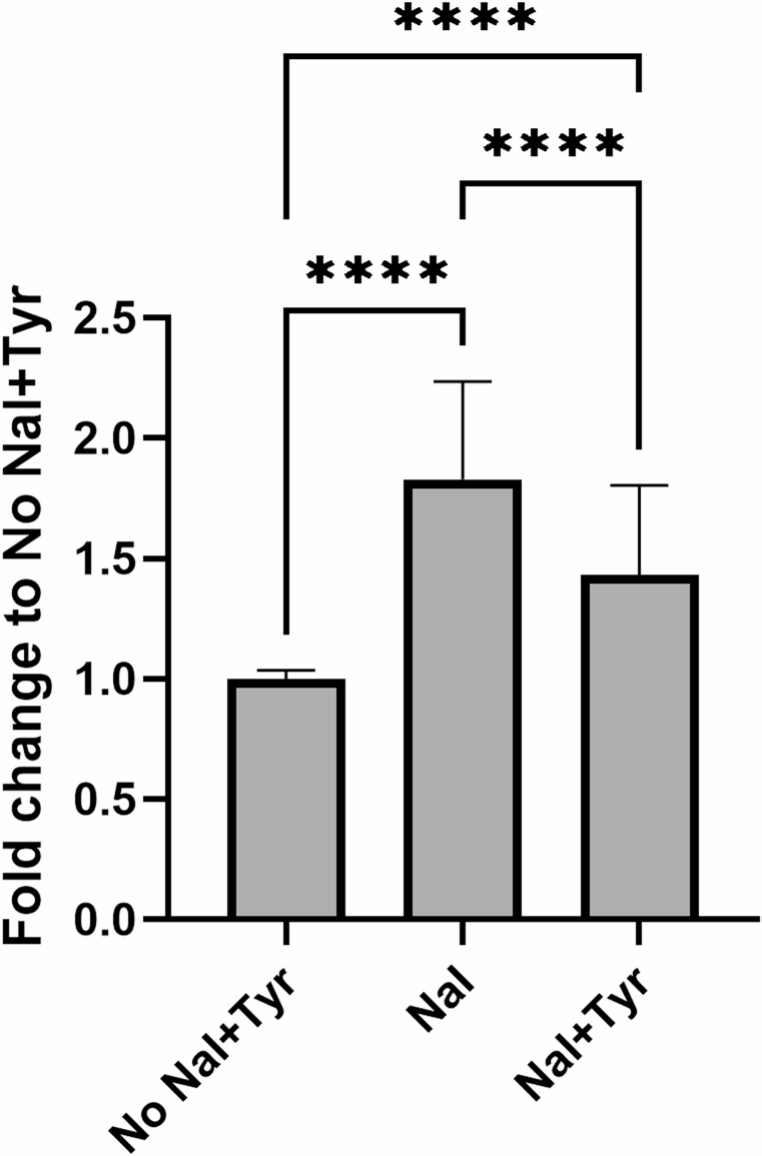
Signal intensity fold change using TPO-containing cell lysate (HEK-TPOA7) in different conditions assessed by the AUR assay. No NaI + Tyr–classical setup of AUR assay; NaI–AUR setup supplemented with 500 µM NaI (final concentration). NaI + Tyr–setup supplemented with 500 µM NaI and 500 µM L-tyrosine (final concentrations). After adding NaI, an increase in intensity was observed. Then, after adding L-tyrosine, the signal decreased similarly to the signal without NaI and L-tyrosine. Average data from nine independent experiments + SD

Upon adding NaI to the AUR assay, a consistent increase in fluorescence intensity compared to the standard assay variant was observed in the experiments with the non-denatured cell lysate (Figs. 2b, 3), suggesting that iodide addition can enhance TPO’s oxidation activity. Interestingly, the enhancement occurred already at the lowest tested NaI concentration (37.5 µM) and did not significantly increase at greater NaI levels (Fig. 2b). Compared to the mean iodine concentration in the thyroid gland, 1.31 ± 0.46 mg/mL (around 10 mM for iodide) (Li et al. 2020), the iodide concentration enhancing the TPO oxidative activity was significantly lower. Since there was a minimal difference in fluorescence signal at higher NaI concentrations, 500 µM NaI was used for testing the chemicals in our study for direct comparability of the results with the Tyr-I assay using the same concentration.

While the addition of NaI increased fluorescence intensity, adding also L-tyrosine significantly reduced the signal (Fig. 3). This indicates that tyrosine may indirectly affect the Amplex UltraRed (AUR) reaction in a system containing TPO and H₂O₂, by altering the substrate preference of TPO. Added tyrosine can act as a sink for oxidized iodine, accelerating the iodination cycle and thereby increasing the consumption of H₂O₂ via TPO. This could lead to less H₂O₂ available for AUR oxidation, potentially reducing the AUR fluorescence signal.

The lysate prepared from the human transfected cell line HEK-TPOA7 overexpressing TPO was used in the standard AUR assay and its modifications with NaI and L-tyrosine. When comparing the data from the AUR assay without NaI to the data from the assay with NaI, several notable observations can be made. In the AUR assay with NaI (AUR + NaI assay), BP2, BPA, MMI, PTU, RSC, SMX, T3, TBBPA, and TCS exhibited significant peroxidase activity inhibition, enabling quantification of IC_50_ values (Table 1). All chemicals that had quantifiable IC_50_ values in the assay without NaI also showed detectable IC_50_ values with NaI. BP2 and RSC were again identified as the strongest TPO inhibitors with comparable IC_50_ values (0.586 µM and 0.394 µM, respectively) as in the original AUR assay, which is, however, significantly higher than those detected in the Tyr-I assay. Comparable IC_50_ values in both AUR assay variants were also observed for BPA, MMI, PTU, and TBBPA. However, T3 and SMX demonstrated quantifiable IC_50_ values in the assay with NaI, but not without NaI, indicating their stronger inhibitory effects on TPO activity in the setup with NaI. Also, TCS exhibited a lower IC_50_ value in the assay with NaI, showing its stronger inhibition in the presence of iodide.

Importantly, AMP, IOP, ETU, and PFOS showed lower yet significant inhibition of fluorescence (≥  20%) in the addition of NaI, although these effects were not detected with the original version of the AUR assay. These chemicals were thus newly recognized by the AUR + NaI assay as TPO inhibitors, which is in congruence with the data from the Tyr-I assay. Around 20% borderline inhibition was detected in the addition of NaI, also for the maximal tested concentration (200 µM) of PFOA, which did not cause significant Tyr-I inhibition.

From these results, we can find that 9 of 21 chemicals showed strong inhibition (over 50%) in the assay with NaI, and 7 of 21 chemicals showed strong inhibition in the assay without NaI. If using a  ≥  20% inhibition threshold as the criterion for TPO inhibitors, 14 of 21 chemicals were identified as TPO inhibitors in the assay with NaI, and 8 of 21 were identified as TPO inhibitors in the assay without NaI. This indicates that more chemicals exhibit TPO inhibition capability in the presence of NaI, suggesting that the assay’s sensitivity is significantly increased by NaI addition. Including iodide not only enhances the oxidation step of TPO activity but also increases the sensitivity of the AUR assay, allowing for the detection of inhibitory effects, namely for rather weak TPO inhibitors that would otherwise be missed.

In the AUR assay with NaI + L-tyrosine using HEK-TPOA7 lysates, BP2, BPA, MMI, PTU, RSC, T3, TBBPA, and TCS exhibited marked TPO inhibition, enabling quantification of IC_50_ values. BP2 and RSC were again the most potent inhibitors. Only IOP and SMX showed lower but significant inhibition (≥  20%) (Table 1).

A similar pattern of results is observed when comparing the data from the AUR assay with added NaI + L-tyrosine with the standard AUR assay. The dose–response curves and effective values were largely comparable for these two assay variants, except for T3 and IOP, with a stronger effect in the variant with added L-Tyr. These similarities document that the addition of L-tyrosine is neither necessary nor beneficial for the assay’s functionality and sensitivity. Moreover, the addition reduces the overall fluorescence signal intensity compared with the variant with NaI only. Although L-tyrosine is a real substrate of the TPO reaction, it is not required to measure peroxidation-dependent inhibition. Therefore, omitting L-tyrosine from the AUR assay with NaI simplifies the protocol and assures the increased sensitivity caused by NaI addition.

Consistent with the results from the Tyr-I assay, seven compounds were identified as having no effect on TPO activity across all AUR assay variants. When comparing data from the AUR assay with NaI to that from the Tyr-I assay, all clear inhibitors demonstrated stronger inhibition in the Tyr-I assay, indicating that it also has higher sensitivity. Nonetheless, with a ≥ 20% inhibition cutoff for identifying TPO inhibitors, 8 of 21 chemicals were identified in the AUR assay, 14 chemicals were identified in the AUR + NaI assay, and 14 of 21 chemicals were identified in the Tyr-I assay. Importantly, the AUR + NaI assay variant revealed the TPO-inhibiting activity of all chemicals that inhibited MIT formation in the Tyr-I assay (though mostly with greater effective concentrations), except for T4, which nevertheless caused only 27% mean MIT production inhibition, and its inhibition was limited only to the greatest tested concentrations in the Tyr-I assay. AMP and ETU exhibited significantly different dose–response profiles between the AUR assays and the Tyr-I assay (Fig. 1). Despite its strong effect with low effective concentrations in the Tyr-I assay, ETU caused only 23% inhibition in the AUR + NaI assay at greatest tested concentration (200 μM). This divergence may be attributed to ETU’s specific mode of action, which inhibits TPO through enzymatic oxidation and iodine trapping (iodination step). Research indicates that ETU-mediated inhibition of TPO-catalyzed reactions occurs only in the presence of iodide, which is accompanied by oxidative metabolism, producing imidazoline and bisulfite ions (Doerge & Takazawa 1990; Freyberger & Ahr 2006). This suggests that ETU requires iodide to exert its effect, indicating direct interaction with TPO during the iodination reaction. Functioning as an alternative electron donor (co-substrate) for TPO’s peroxidase activity, ETU undergoes oxidation by the TPO–iodide complex, thereby preventing the normal transfer of iodide to tyrosine. However, studies by Dong et al. (2024) and Marinovich et al. (1997) reported 54.7% inhibition at 87.5 μM in the AUR assay and approximately 50% inhibition at 50 μM in the Guaiacol assay using lysates from hTPO cell lines, respectively. Notably, these two assays assess only the peroxidation step. If a ≥ 20% inhibition threshold is applied, ETU is qualified as a TPO inhibitor when tested with the AUR with iodide in our study. However, using an iodide-free AUR setup, ETU did not reach this threshold, possibly due to differences in TPO-expressing cell lines or exact assay setup.

### TPO inhibition assessment comparability across various TPO sources and assays

We have compared our data to those reported in previous studies using different models with the AUR assay (Table 1). For human models, Dong et al. (2020, 2024) and ToxCast (US EPA 2024) employed different human TPO-transfected cell lines combined with the AUR assay (only data for MMI available in ToxCast), and ex vivo rat microsomes were used as TPO source by Friedman et al., (2016) and ToxCast (US EPA 2024). However, the TPO inhibition potency was expressed as AC_50_ instead of IC_50_ in these studies. We re-evaluated available data from ToxCast to provide IC_50_ values for direct comparison (Table 1). Eight chemicals were identified as TPO inhibitors using human models in the AUR assay in Dong et al., (2020, 2024). Of these, our IC_50_ values were comparable to data from the literature for four chemicals (BP2, PTU, RSC, TCS); two showed higher IC_50_ values (BPA, MMI), while ETU passed the 20% effect threshold only in the AUR with NaI variant in our study (Table 1). This variability may be due to differences in transfected cell lines used as TPO source, deviations in assay setup, and also a difference between IC_50_ and AC_50_ metrics used in the literature. Only MMI was tested by ToxCast in the human TPO cell line, with a lower IC_50_ value compared to our data. When comparing data obtained with rat thyroid microsomes from ToxCast (US EPA 2024) and Friedman et al. (2016) to our results from the standard AUR assay, five of the nine active chemicals showed similar results (BP2, BPA, PFOA, TBBPA, TCS) and four chemicals had lower effective concentrations in rat TPO (ETU, MMI, PTU, and RSC), and PFOA was consensually inactive. The IC_50_ levels of positive chemicals in our AUR assays (including the variant with NaI addition) are mostly higher than the IC_50_ from the iodination assay with UPLC-MS/MS detection based on human TPO (Reinen et al. 2024), which is consistent with the conclusion from the comparison between the AUR assays and the Tyr-I assay with ICP-MS detection in our study.

### The applicability of different methods for TPO inhibition assessment

The AUR assay supplemented with NaI represents a high-throughput approach suitable for rapid screening of large sample sets. In contrast, the Tyr-I assay has low throughput, higher costs, and requires more time and specialized analytical instrumentation. A combined testing strategy can be employed to balance efficiency with analytical depth. Initially, the AUR assay with NaI could be used as a rapid screening tool, applying a 20% inhibition threshold to identify potential TPO inhibitors. Subsequently, the Tyr-I assay can be applied to the positive hits from the AUR assay to obtain more reliable and detailed information. By integrating both assays, researchers can capitalize on the strengths of each method. The AUR assay enables efficient preliminary screening, while the Tyr-I assay provides a more sensitive and specific follow-up analysis, thereby enhancing the overall reliability and comprehensiveness of TPO inhibition studies.

However, it is important to emphasize that even when combined with NaI, the AUR assay only evaluates the first oxidation step of TPO-mediated activity. As a result, it may fail to detect certain TPO inhibitors, leading to false negatives. Therefore, this assay is more suitable for screening applications, particularly in studies involving complex environmental mixtures, where its simplicity and high-throughput capacity are advantageous.

Thus, this combined strategy is not suitable for identifying thyroid hormone system-disrupting chemicals (THSDCs) that interfere with TPO-mediated iodination or MIT/DIT coupling, but not with peroxidation. For example, ETU was not clearly identified as a TPO inhibitor even with the NaI-enhanced AUR assay. Tyr-I assay incorporates also the iodination step and thus is currently the more appropriate choice for hazard identification of THSDCs, namely for the purposes of regulatory testing of chemicals. Nevertheless, the current Tyr-I assay setups cover only two of the three TPO enzymatic activities, excluding MIT/DIT coupling. Further assay modifications would be necessary to detect THSDCs affecting this third activity.

To our knowledge, there is only a single study that demonstrated the detection of TPO inhibition with rat thyroid microsomes by complex environmental samples from both surface and treated wastewater (Leusch et al. 2018). Notably, the effect was detected at environmentally relevant concentrations, at least in the case of the treated wastewater sample. This finding indicates that TPO inhibition could be relevant for aquatic organisms exposed to the environmental pollutant mixtures.

Supporting this, a wide range of studies have detected chemicals identified as TPO inhibitors using ex vivo rat thyroid microsomes in various environmental matrices (Friedman et al. 2016). These include compounds from diverse chemical groups, such as polycyclic aromatic compounds, bisphenols, and current-use pesticides. These compounds have been found in matrices such as surface waters (Novák et al. 2018; Sauer et al. 2023), house dust (Nováková et al. 2022; Pinto-Vidal et al. 2024), indoor air (Nováková et al. 2022), and ambient air (Nováková et al. 2020). Even though the detected concentrations of these chemicals were often relatively low, they are typically part of complex mixtures containing many other compounds for which the TPO inhibition potential is unknown. This complexity highlights the necessity of applying high-throughput methods for assessing higher numbers of complex samples and relevant exposure mixtures and prioritizing the most relevant for further detailed characterization. Namely for these purposes the employment of (iodide amended) AUR screening assays might be a viable cost-effective solution.

## Conclusion

The HEK-TPOA7 cell line was successfully utilized as a TPO source in the Tyr-I assay, demonstrating greater sensitivity than the AUR assay for detecting TPO inhibition. The addition of sodium iodide enhanced the sensitivity of the AUR assay. For future applicability of the iodide-supplemented AUR assay, the concentration of added iodide should be optimized for the best sensitivity, and appropriate controls with heat-inactivated lysate should always be included to address possible interference with the TPO non-specific oxidation of the fluorescent substrate. Importantly, the Tyr-I assay demonstrated not only the highest sensitivity but also consistent replicability, reinforcing its value relative to comparable methods in recent literature.

The findings document that chemicals from diverse use groups and structural classes—including widespread environmental contaminants—exhibit TPO inhibition across different assay types. This underscores the relevance of TPO inhibition as a mode of action linked to various adverse effects and highlights the urgent need for efficient assessment tools.

By employing a complementary approach that integrates the AUR assay with sodium iodide for high-throughput screening, followed by the more sensitive Tyr-I assay for detailed analysis, researchers can improve the overall efficiency of TPO inhibition screening studies, e.g., with higher numbers of environmental samples or complex environmental mixtures, where the low-throughput, more expensive Tyr-I assay is not feasible. However, since the AUR assay covers only the oxidation step catalyzed by TPO and does not identify THSDCs affecting the iodination step, applications requiring mechanistic insight or direct regulatory relevance, namely when characterizing overall TPO inhibition by chemicals for decision making, should prioritize the Tyr-I assay, despite its lower throughput, due to its greater physiological relevance.

## Supplementary Information

Below is the link to the electronic supplementary material.

Supplementary Material 1.
