# Supplementary material for: In vitro thyroperoxidase inhibition assessment by LC-ICP-MS-based L-tyrosine iodination assay: comparison with Amplex Ultrared assay and its modifications
Source: Arch Toxicol. 2026 Jan 14;100(4):1485–99. doi: 10.1007/s00204-025-04258-y (PMC13043538; doi:10.1007/s00204-025-04258-y)
Supplement: Supplementary file 1 — Supplementary Material 1. [file 204_2025_4258_MOESM1_ESM.docx]

**Supplementary Materials** to the paper:

## ***In vitro thyroperoxidase inhibition assessment by LC-ICP-MS-based L-tyrosine iodination assay: comparison with Amplex Ultrared assay and its modifications***

## Runze Liu, Jiří Novák, Jan Kuta, Marie Smutná, Klára Hilscherová*

## Masaryk University, Faculty of Science, RECETOX, Kamenice 753/5, 625 00 Brno, Czech Republic

## *Corresponding author: [klara.hilscherova@recetox.muni.cz](mailto:klara.hilscherova@recetox.muni.cz)

## **Tyr-I assay optimization**

The procedure was based on a previously described method (Tater et al. 2021) with modifications. The assay was optimized using rat thyroid microsomes (RM) and further with the lysate of the TPO-expressing HEK-TPOA7 cell line. The effect of protein concentration on the reaction was evaluated, and the optimal cell lysate protein level was determined to be 0.12 µg/µL (Figure S1), which elicited significant TPO activity in HEK-TPOA7, whereas the control lysates without active TPO (denatured HEK-TPOA7 and HEK293T) completely inhibited the non-enzymatic MIT production. Thus, there was no difference between denatured HEK-TPOA7 and HEK293T cell lysates, serving as the baseline control. Two concentrations of H₂O₂ were tested. The higher peroxide concentration (100 µM) led to an increase in spontaneous iodination of L-tyrosine in the absence of cell homogenate (Figure S2). However, at 40 µM H₂O₂, the spontaneous iodination product level was below the LOQ and significantly lower than in the presence of native TPO. Therefore, 40 µM H₂O₂ was selected for the final assay setup.

In addition to L-Tyr as the TPO substrate, the iodination reaction was also tested with monoiodotyrosine (MIT) and diiodotyrosine (DIT), yielding DIT and thyroxine (T4; Figure S3). L-Tyr to MIT conversion was prioritized in this study because it resulted in the highest product yields and enabled reliable detection of L-Tyr iodination inhibition using the analytical method.

While non-enzymatic iodination of the substrate was detected in the absence of any protein, it was either significantly reduced or completely inhibited in the presence of heat-inactivated TPO-containing cell lysate or cell lysate from non-transfected HEK293T cells with 40 µM H₂O₂ (Figures S2, S3). In contrast, replacing TPO with the same protein concentration of bovine serum protein did not affect iodination (data not shown). This suggests that reaction inhibition may have been influenced by other factors, such as cell membranes, rather than by proteins alone. The final assay setup included a 60-minute exposure time, as it provided sufficient product yields with relatively low variability (exposure times tested: 30, 60, 120 min; see Figure S3).

Figure S1. TPO-catalyzed L-Tyr iodination reaction – effect of protein concentration of cell lysates with native TPO (HEK-TPOA7) and without active TPO (non-transfected HEK293T cells, denatured HEK-TPOA7. The analytical limit of quantification (LOQ) for MIT and DIT was 0.016 and 0.012 µg/µL, respectively. DIT was detected around LOQ at the highest protein concentrations of native HEK-TPOA7 cell lysate (data not shown). Average data from three independent experiments with 40 µM H₂O₂.

Figure S2. Comparison of MIT produced in L-Tyr iodination reaction with 40 and 100 µM H_2_O_2_ in the presence of native TPO (HEK-TPOA7), denatured TPO (HEK-TPOA7-denat – heat-inactivated HEK-TPO-A7 cell lysate), non-transfected HEK293T cell lysate (all lysates at protein concentrations 0.12 µg protein/µL), and in buffer without protein (protein-free control). Higher peroxide concentration significantly increased spontaneous iodination of L-Tyr in protein-free control and slightly in the control with denatured HEK-TPOA7 (on average 0.19 µM; 6% of the native HEK-TPOA7 activity with 100 µM H_2_O_2_), while it was below LOQ in the variants without active TPO (denatured HEK-TPOA7, HEK293T) and 40 µM H_2_O_2_. Average data from three independent experiments using the final optimized setup with 60 min incubation.

Figure S3. TPO-catalyzed conversion of MIT (600 µM) to DIT and DIT (540 µM) to T4 (which could not be distinguished from rT3 analytically in a short 5 min run, distinguishing is not a problem with a longer run; LOQ 51 nM for T4+rT3) in the presence of native TPO (RM - rat thyroid microsomal fraction), and in buffer without protein (protein-free control). T4 was not detected in MIT-DIT conversion. Although the reaction products were detected, their concentrations were relatively low.

Figure S4. AUR assay performance in controls without iodide, and in combination with iodide (500 µM) and iodide + L-tyrosine (both 500 and 500 µM). Fluorescence data expressed relative to protein-free control without iodide and L-tyrosine (PBS). HEK-TPOA7-denat – heat-inactivated cell homogenate from TPO-expressing cell line, HEK293T – native homogenate of the cell line without TPO transfection; HEK-TPOA7 – native homogenate of TPO-transfected HEK293T cell line. Data from three independent experiments.

## **Results**

Figure S5. Comparison of dose-response curves for chemicals with no significant inhibitory effects across different assay set ups in human transfected cell line HEK-TPOA7. Relative TPO activity rate (%): The signal intensity from the well-containing cell lysate and chemical was background-corrected (average signal intensity of boiled lysate wells), then normalized to the solvent control (100% activity).

Figure S6. Dose-response curves for tested chemicals with Tyr-I assay in rat microsomes. Relative TPO activity rate (%): The signal intensity from the well-containing rat microsomes and chemical was normalized to the solvent control (100% activity; data from three independent experiments).

Table S1. Model chemicals employed in the study, the chemical type category, and tested concentration ranges. AUR - all AUR assays. Tyr-I - Tyrosine iodination assay.

| Abbrev. | Chemical | CAS No. | Chemical Type Category | Tested concentration range (µM)  (AUR)/(Tyr-I) |  |
| --- | --- | --- | --- | --- | --- |
| AMP | Ampicillin | 2795-39-3 | Antibiotics and Antimicrobials | 0.2-200/3.13-200 |  |
| BP2 | 2,2′-4,4′-tetrahydroxy benzophenone | 69-53-4 | Personal Care and Consumer Products | 0.2-200/0.004-0.1 |  |
| BPA | Bisphenol A | 131-55-5 | Industrial Chemical | 0.2-200/0.09-10 |  |
| CBZ | Carbamazepine | 51-52-5 | Pharmaceuticals | 0.2-200/7.41-200 |  |
| DBP | Dibutylphthalate | 108-46-3 | Industrial Chemicals | 0.2-200/7.41-200 |  |
| DON | Deoxynivalenol | 69-72-7 | Mycotoxins | 0.2-200/3.13-200 |  |
| ETU | Ethylene thiourea | 723-46-6 | Industrial Chemical | 6.25-200/0.01-10 |  |
| IOP | Iopanoic acid | 55-06-1 | Pharmaceuticals | 0.2-200/7.41-200 |  |
| MMI | Methimazole | 80-05-7 | Pharmaceuticals | 0.02-200/0.03-3.33 |  |
| PCL | Perchlorate | 51-48-9 | Industrial Chemicals | 0.2-200/7.41-200 |  |
| PFOA | Perfluorooctanoic acid | 298-46-4 | Perfluorinated Chemicals | 0.2-200/7.41-200 |  |
| PFOS | Perfluorooctane sulfonate | 84-74-2 | Perfluorinated Chemicals | 0.2-200/7.41-200 |  |
| PTU | 6-propylthiouracil | 51481-10-8 | Pharmaceuticals | 0.0002-200/0.16-20 |  |
| RSC | Resorcinol | 96-45-7 | Personal Care and Consumer Products | 0.002-200/0.004-0.05 |  |
| SA | Salicylic acid | 79-94-7 | Personal Care and Consumer Products | 0.002-200/7.41-200 |  |
| SMX | Sulfamethoxazol | 96-83-3 | Antibiotics and Antimicrobials | 0.2-200/2-100 |  |
| T3 | 3,3′,5-Triiodo-L-thyronine | 60-56-0 | Thyroid Hormone | 0.31-10/0.31-10* |  |
| T4 | 3,3′,5,5″-Tetraiodo-L-thyronine | 3380-34-5 | Thyroid Hormone | 0.2-200/7.41-200 |  |
| TBBPA | Tetrabromobisphenol A | 7601-89-0 | Industrial Chemicals | 0.1-200/0.063-10 |  |
| TCS | Triclosan | 335-67-1 | Antibiotics and Antimicrobials | 0.2-200/ 1.56-200 |  |
| TPP | Triphenyl phosphate | 115-86-6 | Industrial Chemicals | 0.2-200/7.41-200 |  |

* For Tyr-I assay with rat microsomes, the highest tested concentration was 5 µM.

**Reference**

Tater, A., Gupta, A., Upadhyay, G., Deshpande, A., Date, R., Tamboli, I.Y., 2021. In vitro assays for characterization of distinct multiple catalytic activities of thyroid peroxidase using LC-MS/MS. Curr. Res. Toxicol. 2, 19–29. https://doi.org/10.1016/j.crtox.2021.01.001
